# Heritability and Genome-Wide Association Studies for Hair Color in a Dutch Twin Family Based Sample

**DOI:** 10.3390/genes6030559

**Published:** 2015-07-13

**Authors:** Bochao Danae Lin, Hamdi Mbarek, Gonneke Willemsen, Conor V. Dolan, Iryna O. Fedko, Abdel Abdellaoui, Eco J. de Geus, Dorret I. Boomsma, Jouke-Jan Hottenga

**Affiliations:** Department of Biological Psychology, VU University, Amsterdam 1081 BT, The Netherlands; E-Mails: b.lin@vu.nl (D.B.L.); h.mbarek@vu.nl (H.M.); a.h.m.willemsen@vu.nl (G.W.); c.v.dolan@vu.nl (C.V.D.); i.o.fedko@vu.nl (I.O.F.); a.abdellaoui@vu.nl (A.A.); eco.de.geus@vu.nl (E.J.G.); d.i.boomsma@vu.nl (D.I.B.)

**Keywords:** hair color, twin-family based heritability, GRM based heritability, Genome Wide Association Study

## Abstract

Hair color is one of the most visible and heritable traits in humans. Here, we estimated heritability by structural equation modeling (N = 20,142), and performed a genome wide association (GWA) analysis (N = 7091) and a GCTA study (N = 3340) on hair color within a large cohort of twins, their parents and siblings from the Netherlands Twin Register (NTR). Self-reported hair color was analyzed as five binary phenotypes, namely “blond *versus* non-blond”, “red *versus* non-red”, “brown *versus* non-brown”, “black *versus* non-black”, and “light *versus* dark”. The broad-sense heritability of hair color was estimated between 73% and 99% and the genetic component included non-additive genetic variance. Assortative mating for hair color was significant, except for red and black hair color. From GCTA analyses, at most 24.6% of the additive genetic variance in hair color was explained by 1000G well-imputed SNPs. Genome-wide association analysis for each hair color showed that SNPs in the MC1R region were significantly associated with red, brown and black hair, and also with light *versus* dark hair color. Five other known genes (*HERC2*, *TPCN2*, *SLC24A4*, *IRF4*, and *KITLG*) gave genome-wide significant hits for blond, brown and light *versus* dark hair color. We did not find and replicate any new loci for hair color.

## 1. Introduction

Hair color is a genetic and physiologically complex phenotype, which represents one of the most visible variations within humans and between populations [1]. It influences many human interactions including spouse selection. Previous studies of the effect of hair color on social behavior and personality have shown that hair color is related to attractiveness, but it is unclear how strong this effect is [2,3]. In general, individuals with a lighter hair color, e.g., blond, have a higher probability to receive courtship solicitations, as compared to individuals with red hair. Dyeing the hair has been practiced for over four thousand years, and is still immensely popular today, with over 75 percent of American women dyeing their hair [4].

Pigmentation of skin and hair is important for protection against sunlight [5]. From a medical point of view, pigmentation is also relevant, as the mechanisms underlying pigmentation are involved in one of the most aggressive types of cancer, namely malignant melanomas. These tumors of melanocytes, cause about 75% of deaths related to skin cancer, with high rates of incidence in Caucasians, especially in northwestern Europeans [6]. This prevalence is associated with ultraviolet light (UV) exposure and the amount of skin pigmentation [7].

Hair pigmentation is a highly heritable trait. In Europeans, genetic factors explain a large part (61%–92%) of the variation in natural hair color, while the rest of the variation is due to environmental influences and measurement error [8,9,10]. Previous studies have found several genes that are relevant in human pigmentation, especially in the melanosome biogenesis or the melanin biosynthetic pathways [11]. Differences in eye and hair color are mainly due to variation in the amount, type, and the packaging process of key pigment molecule melanin polymers produced by melanocytes secreted into keratinocytes. Melanin exists in two basic forms: brown-black eumelanin and yellow-red pheomelanin. Hair color mainly depends on the ratio of eumelanin and pheomelanin [12].

Studying the genetic background of a human pigmentation trait like hair color is useful to understand human evolution and biology and may have important applications to melanoma treatment and to forensics. Loss-of-function mutations at the melanocortin 1 receptor (*MC1R*) are known to be associated with a switch from eumelanin to pheomelanin production, resulting in a red or yellow coat color in animal models [13]. Over 30 MC1R variant alleles correlated with skin and hair color have been identified [14]. In addition to MC1R, the HERC2 and OCA2 genes, located close to the MC1R gene on chromosome 15, are also related to hair color. Studies have shown that HERC2 regional variants function as enhancers regulating OCA2 transcription [15]. Variants located in and around these genes determine the normal human hair pigment variation.

To identify human pigmentation genetic variants and to gain new knowledge of genetic background of hair color in the Dutch population, we estimated the broad-sense heritability of hair color with a liability threshold model on twins and their family members from the Netherlands Twin Register (NTR) [16]. In addition, we estimated SNP heritability based on the measured and imputed SNP data from unrelated NTR individuals, by associating their genetic relatedness to their phenotypic resemblances using GCTA [17]. Finally, to identify genetic variants underlying the heritability of hair color, we performed a series of genome-wide association studies (GWAS) of hair color in this population-based sample.

## 2. Materials and Methods

### 2.1. Subjects

Hair color data were available for 25,201 NTR participants, clustered in a total of 7862 families, with ages ranging between 14 and 80 years old. Each participant completed one or more longitudinal surveys that included questions about age, sex, and natural hair color. The self-reported hair color was obtained from a question with five answer possibilities: “fair/blond”, “light brown”, “red/auburn”, “dark brown”, and “black”. Written informed consent was obtained from all participants. The Medical Ethics Committee of the VU University Medical Center approved the study protocols.

For the twin-family based heritability analyses, we selected families comprising at most 6 members (N = 20,142). The subjects included in these families were the twins (N = 15,359), a maximum of two siblings (N = 2008), and the biological father and mother (N = 2774) (Table S1). Of the twins, 2345 were monozygotic males (MZM), 4651 were monozygotic females (MZF), 1678 dizygotic males (DZM), 2710 were dizygotic females (DZF) and 3975 were from an opposite-sex (DOS) twin pair (Table S2). For the GWAS analyses, a total number of 7091 related subjects were available with genotype, phenotype and covariate data (Table S3). For GCTA, only unrelated people were selected from the available GWAS sample, resulting in a sample of 3340 individuals.

### 2.2. DNA Sampling and Genotyping

Buccal or blood DNA samples (N = 14,003) were collected for multiple NTR projects. DNA extraction and purification of these samples were performed at various points in time, following several manufacturer specific protocols to obtain the best quality and concentration prior to SNP platform genotyping [17]. Genotyping of several partly overlapping subsets was done on multiple platforms. Chronologically the following platforms have been used Affymetrix Perlegen 5.0, Illumina 370, Illumina 660, Illumina Omni Express 1M, and Affymetrix 6.0. After array specific data analysis, genotype calls were made with the platform specific software (BIRDSUITE, APT-GENOTYPER, BEADSTUDIO).

Quality control was done within and between platforms and subsets prior to imputation. For each platform, the individual SNP markers were lifted over to build 37 (HG19) of the Human reference genome, using the LiftOver tool. SNPs that were not mapped at all, SNPs that had ambiguous locations, and SNPs that did not have matching (or strand opposite alleles) were removed. Subsequently, the data were strand aligned with the 1000 Genomes GIANT phase1 release v3 20,101,123 SNPs INDELS SVS ALL panel. SNPs from each platform were removed if they still had mismatching alleles with this imputation reference set, if the allele frequencies differed more than 0.20 with the reference. From each platform, SNPs with a Minor Allele Frequency (MAF) <0.01 were removed, as well as SNPs that were out of Hardy–Weinberg Equilibrium (HWE) with *p* < 0.00001. Samples were excluded from the data if their expected sex did not match their genotyped sex, if the genotype missing rate was above 10% or if the Plink F inbreeding value was either >0.10 or <−0.10.

After these steps, the data of the individual arrays were merged into a single dataset using PLINK 1.07 [18]. Within the merged set, identity by state (IBS) sharing was calculated between all possible individual pairs and compared to the known family structure of the NTR study. Samples were removed if the data did not match their expected IBS sharing. DNA samples, which were typed on multiple platforms, were tested to ascertain that the concordance rate among overlapping SNPs exceeded 99.0%. If the concordance rate was lower, we removed all data of these samples. Subsequently, from each MZ twin pair a single DNA sample was selected. The HWE-, MAF- and the reference allele frequency difference <0.20 filters were re-applied in the combined data. As a final step, SNPs with C/G and A/T allele combinations were removed when the MAF was between 0.35 and 0.50 to avoid incorrect strand alignment.

Phasing of all samples and imputing cross-missing platform SNPs was done with MACH 1 [19,20]. The phased data were then imputed with MINIMAC [21] in batches of around 500 individuals for 561 chromosome chunks obtained by the CHUNKCHROMOSOME program [22]. After imputation, DNA confirmed MZ twins were re-duplicated back into the data. The format of the data was transformed to the basic three probabilities SNPTEST gen.gz format, as this is the most general applicable format for the subsequent genomic analyses tools. The mean imputation quality R^2^ metric is 0.38 (based on all 30,051,533 imputed autosomal SNPs).

After imputation, SNPs were filtered based on the Mendelian error rate in families. The Mendelian error rate was calculated on the best guess genotypes in families (trios and sib-pairs with parents) using first GTOOL to calculate best guess genotypes and then PLINK 1.07 to analyze the data. SNPs were removed if the Mendelian error rate >2%, if the imputed allele frequency differed more than 0.15 from the 1000G reference allele frequency, if MAF < 0.005 and if R^2^ < 0.30. HWE was calculated on the genotype probability counts for the full sample, and SNPs were removed if the *p*-value < 0.00001. This left 7,981,681 SNPs prior to the statistical analyses.

### 2.3. Statistical Analyses

First, we created binary variables for each hair color, representing a given hair color *versus* the other hair colors. In the following analysis, we found a much lower heritability for the light brown and dark brown classification than for other classifications. By combining light and dark brown hair color into a single brown category, we obtained a more sensible heritability estimate as compared to the other colors. Assuming this discrepancy is due to confusion among participants concerning the distinction light *versus* dark brown, we decided to proceed with a simple category “brown”. An additional binary variable, denoted “light *versus* dark”, was created representing blond and red hair *versus* brown (light and dark) and black. We studied this binary variable to detect the genes involved in the switch from eumelanin to pheomelanin. Hence, in total we analyzed the following 5 binary classifications, namely “blond *versus* non-blond”, “red *versus* non red”, “brown *versus* non-brown”, “black *versus* non-black”, and “light *versus* dark”.

### 2.4. Genetic Covariance Structure Modeling of Hair Color

To obtain an indication of family clustering, we calculated (tetrachoric) correlations among family members for binary hair color variables in OPENMX [23]. We adopted a liability-threshold model, in which the probability of having a given hair color (say blond) is a function on the position on the continuous (standard normal) liability scale. The tetrachoric correlations express the association between family members at the level of the liability. The actual proportion of the given hair color in the sample is expressed in terms of the threshold, *i.e.*, a point on the liability scale. For instance, a threshold of zero on the standard normal liability scale is associated with a proportion of 0.50 (e.g., 50% are blond). The more extreme the threshold is, the greater or smaller the proportion (threshold of 1 implies ~84% are blond; a threshold of −1 implies ~16% are blond).

OPENMX was then used to estimate the heritability of hair color by fitting a genetic model to the liabilities of the parents and offspring (twins and sibs). The total liability variance was decomposed into genetic and environmental variance components [24]. We started with a model, which included the following effects: (1) the spousal correlation to account for phenotypic assortative mating (see supplemental material); (2) quantitative sex differences in the genetic and genetic influences on the liability; and (3) age effects on the threshold. Our visual inspection of the tetrachoric correlations strongly suggested the absence of common (or shared) environment influences for the hair colors blond, brown, and the dark *versus* light dichotomy [25] (*i.e.*, the MZ correlation was higher than twice the DZ correlation). The twin correlations for the red and black hair color suggested the possible presence of common environmental influences (C). We focused on the model including additive genetic effects (A), which represent the sum of the additive effects of all loci relevant to the trait, genetic dominance effects (D), which represent interactions between alleles at the same locus, and unique (or unshared) environmental effects (E), which are not shared by family members. Note that the unique environmental factors may include genetic causes, such as personal genetic mutations, and also measurement errors. In the analyses of red and black hair, we also considered the model including C instead of D. We accommodated the phenotypic assortative mating by deriving the expected correlation between the family members taking into account phenotypic assortment (more detail in Supplementary Material 1). Since the prevalence of hair color may vary with age, we included age (Z transformed) as covariates on the liability threshold. We fitted the full model as described, and tested various effects by dropping the effects and conducting a likelihood ratio test. In this fashion we tested whether the prevalence of hair color varied with age, whether the spousal correlation was zero (*i.e.*, random mating for hair color), whether sex effects in genetic architecture were absent, and whether dominance effects (or common environmental effects in the cases of red and black hair) were absent. Given the power that our large sample size confers to detect minor effects, we tested the specific effects mentioned using a stringent alpha of 0.0001 [26].

Since hair color displays a geographical gradient in the Netherlands (e.g., blond is more prevalent in the northern provinces), we refitted the full twin model in the subsample of genotyped individuals (5777 individuals in 2225 families) while correcting for the gradient using their data on three Dutch genotype principal components (PCs). These PCs were calculated from the genotype data with the EIGENSOFT software [27]. First, 10 PCs were calculated by projecting the NTR data on the 1000 Genomes reference set and all individuals with non-Dutch ancestry were excluded. Then, three new only Dutch PCs within the NTR samples were calculated, which highly correlate with the geographic location [28]. We examined if these PCs affected the broad heritability with blond, brown and light *versus* dark hair colors (the rare black and red hair colors excluded) by including these PCs in the ADE model as covariates on the threshold.

### 2.5. Variance Explained by Common SNPs (GCTA)

The proportion of variance of the binary hair color traits that can be explained by the measured and imputed SNPs was estimated in GCTA (Genome-wide Complex Trait Analysis) using the Restricted maximum likelihood (REML) analysis procedure under a case-control design, where we report the proportion of genetic variance explained on the underlying liability scale untransformed for prevalence [17]. Sex and age were included as covariates and the three Dutch PCs were alternated. A single genetic relationship matrix (GRM) was build to test all hair colors. From the 1000 genomes imputed and cleaned data, we selected SNPs with a minimum imputation R^2^ quality metric of 0.80 and MAF > 0.01. In order to avoid explained variance and artificially increased GRM differences due to differing platforms and subsamples, additional SNP Quality Control (QC) included an evaluation of the SNP platform effects. We tested the effect of different platforms and removed SNPs showing platform effects. This was done by defining individuals on a specific platform as cases and the remaining individuals as controls. Allelic association was then calculated and SNPs were removed if the specific platform allele frequencies were significantly different from the remaining platforms with *p*-value < 0.00001. The selected 5,987,253 SNPs were transformed to best guess Plink binary format, and subsets were made for each of the 22 chromosomes. The GRM for all NTR samples was then calculated per chromosome and subsequently the 22 matrixes were merged into a single autosomal GRM using GCTA. Ethnic outliers based on the PCs were excluded and 3340 unrelated individuals with hair color data were selected using the standard GRM cut-off filter of 0.025.

### 2.6. GWA Analyses

The GWA analyses were run for all binary hair color variables. The input SNPs used were all 7,981,681 that passed the initial imputation QC, however post GWAS analyses we additionally filtered the SNPs, depending on the cases sample size for MAF and imputation quality in the sub-sample of individuals with a hair color phenotype. Re-filtering on MAF > 0.01 was done after the GWAS analyses for blond, brown, and light *versus* dark hair color. For red and black hair color a MAF > 0.05 was selected to account for the lower prevalence of these hair colors. For imputation quality we filtered on the Plink information criterion, which is similar to R^2^: the variance of the mean posterior genotype probabilities divided by the maximum expected variance given full HWE and complete known genotypes. In all hair colors we filtered with 0.40 < Plink Info < 1.02. In total 6,473,680 SNPs survived this QC leading to a mean original imputation R^2^ of 0.77 (0.22) for MAF 0.01–0.05 and a mean R^2^ of 0.97 (0.07) for MAF ≥ 0.05 for the selected SNPs.

Since, hair color is affected by population stratification; we used the three Dutch ancestry PCs as covariates [28] and excluded ethnic outliers similar as the twin modeling. Other covariates that were included were binary dummies for genotyping platform, sex and age. Analysis was performed with the PLINK 1.07 software running a logistic regression on each SNP, taking genotype inaccuracy into account using dosage data in the analyses. Because the GWAS data includes family members, we included the family option in our analyses, which takes the familial structure into account using a sandwich estimator [29]. The assortative mating of parents was corrected with the same familial-based correction [30]. Only one of the two monozygotic twins was selected for the GWAS analyses in case there was hair color data for both. For the GWAS, we assume a p-value less than 5 × 10^−8^ to be statistically significant [31].

## 3. Results and Discussion

### 3.1. Prevalences and Phenotypic Tetrachoric Correlations

The prevalences and the familial tetrachoric correlations based on N = 20,142 (clustered in 7497 families) for blond, brown, red, black and light *versus* dark hair colors are presented in Table 1. The prevalence of the blond, brown, and light *versus* dark hair colors ranges from 39% to 53%, but the prevalence of red and black hair colors is appreciably lower (red: 4.5%; black: 3.4%).

The MZ correlations are consistently high, ranging from 0.93 to 0.99. The full sib correlations, including the DZ twins, are lower (range 0.14–0.86) and that also goes for the parent-offspring correlations (range 0.19–0.73). Overall, the pattern of correlations is consistent with the expected large genetic contribution to hair color variation. In all but the red and black hair colors, the correlations suggest the presence of additive genetic and dominance effects, as the MZ correlations (range 0.93–0.99) are appreciably higher than the full sibs (including the DZs, range 0.14–0.49) and the parents and offspring (range 0.33–0.58). The correlations among first-degree relatives for red and black hair color tend to be larger than for the other hair colors. However, given the lower prevalence of these hair colors, these correlations are subject to larger standard errors. The spousal correlations suggest weak assortative mating with respect to hair colors (except black hair where the correlation is −0.179 and red hair where the correlation is 0.528). We note that the spousal correlations may be due to direct phenotypic assortment for hair color, or may be related to the geographic population structure of the Netherlands, as was established previously in our data [28].

### 3.2. Genetic Covariance Structure Modeling

The results of fitting all models are shown in Tables S4 and S5. Briefly, the ADE model optimally fits for the blond, brown and light *versus* dark hair colors. Furthermore, the results unambiguously show that no effects could be dropped, *i.e.*, age effects, phenotypic assortment, sex differences on the genetic architecture, and non-additive genetic effects are present (all *p*-values are <0.0001). The results pertaining to the red and black hair colors are mixed. In the ACE model, as fitted to the red hair color, there is no sex difference (Table S5; *p*-value = 0.05), but all other effects are present (Table S5, *p*-value < 0.0001). With respect to the black hair color, we find a simple AE with age effects, but no assortative mating (*p*-value = 0.07), no C (*p*-value = 0.21) and no sex differences (*p* = 0.56). The absence of specific effects in the red and black hair colors may be due to low power as a consequence of the low prevalence of these hair colors.

Table 2 shows the parameter estimates as obtained in the best fitting model. With respect to the genetic influences, we find that the broad sense heritabilities in the ADE models are high (over 0.90). The narrow sense heritability of red hair color, as obtained in the ACE models, is lower (0.73), and the narrow sense heritability of black hair color is 0.96. Inclusion of the genotype based three Dutch PCs in the models, to account for Dutch genetic population stratification and the geographical gradient of hair color in the Netherlands, did not strongly alter the estimates of heritability, phenotypic assortment and variance decomposition (Table S6). However, the PCs do significantly explain hair color variance in all modeled colors (all *p*-values are <1.0 × 10^−9^).

**Table 1:** Polychoric correlation of hair colors in individuals of the same family for different classification groups.

Classification	Prevalence	rSpouse	rFS	rFD	rMS	rMD	rMZM	rMZF	rDZM	rDZF	rDOS	rBB	rSS	rBS
Blond	0.394	0.226	0.364	0.361	0.454	0.472	0.959	0.972	0.357	0.337	0.454	0.417	0.375	0.442
Brown	0.527	0.144	0.190	0.238	0.351	0.413	0.935	0.967	0.375	0.373	0.416	0.375	0.383	0.421
Red	0.045	0.528	0.576	0.485	0.725	0.593	0.978	0.934	0.477	0.610	0.608	0.406	0.529	0.636
Black	0.034	−0.179	0.334	0.352	0.413	0.274	0.928	0.991	0.706	0.857	0.567	0.143	0.752	0.570
Light *versus* dark	0.439	0.229	0.387	0.380	0.472	0.476	0.957	0.971	0.375	0.398	0.478	0.481	0.407	0.490

Prevalence: the prevalence of the given hair color in our sample (N = 20,142); rSpouse: phenotypic spousal correlation; rFS: father-son correlation; rMS: mother-son correlation; rFD: father-daughter correlation; rMD: mother-daughter correlation; rMZM: monozygotic male twin correlation; rDZM: dizygotic male twin correlation; rMZF: monozygotic female twin correlation; rDZF: dizygotic female twin correlation; rDOS: dizygotic opposite-sex twins correlation; rBB: brothers correlation, rSS: sisters correlation, and rBS: brother-sister correlation.

**Table 2:** The heritability and other parameters estimated from A (D or C) E model for hair color (N = 20,142 individuals).

Classification	M	Am	Dm/Cm	Em	Af	Df/Cf	Ef	h^2^m	h^2^f
Blond (ADE)	0.210	0.39	0.57	0.04	0.73	0.24	0.03	0.96	0.97
Brown (ADE)	0.143	0.22	0.71	0.07	0.68	0.29	0.03	0.93	0.97
Red (ACE)	0.704	0.73	0.26	0.01	0.73	0.26	0.01	0.73	0.73
Black (AE)	0	0.96		0.04	0.96		0.04	0.96	0.96
Light *versus* dark (ADE)	0.208	0.42	0.54	0.04	0.75	0.22	0.03	0.96	0.97

M: phenotypic assortative mating coefficient (as estimated in the model); Am, Af = total additive variance in males and females; Dm, Df = total non-additive variance in males and females (blond, brown and light *versus* dark); Em, Ef = total unique environment variance plus measurement error in males and females; Cm, Cf = shared environment variance in males and females (red and black hair color); h^2^m = heritability in males, h^2^f = heritability in females (broad sense h^2^ of blond, brown, and light *versus* dark; narrow sense h^2^ of red and black).

### 3.3. Variance of Hair Color Explained by Autosomal SNPs Using GCTA

The GCTA analyses in unrelated individuals show that depending on the hair color, at most 24.6% of the hair color liability can be explained by the autosomal SNPs (Table 3). All SNPs of the top individual chromosomes explain between 1% and 16.3% of the liability. These SNPs are on chromosome 16 (where the *MC1R* gene is located) for red hair, on chromosome 15 (where the *HERC2* gene is located) for brown and light *versus* dark, and on chromosome 6 (where the *RPS6KA2* gene is located) for black hair color. We also studied the explained liability given by the top SNPs that are already known hair color loci as reported in earlier studies. These SNPs explain between 0.5% and 6.9% of the hair color liability in our sample.

The estimated genetic liability explained by common SNPs is low (<30%), given the fact that the heritability of hair color as a trait is very high (>70%). There are several possible explanations for this: GCTA is less appropriate for binary data than for quantitative phenotypes; there are not many genes related to hair color; the distribution of effect sizes of the genes and a combination of assortative mating plus possible common environment and dominance for this trait are not accounted for by the modeling assumptions of GCTA. In addition, SNPs were filtered by MAF > 0.01 and R^2^ > 0.80, leading to a large reduction of all SNP variants present within the GCTA matrix, and the coverage of rare alleles and less well imputed SNPs might therefore not be optimal. Also the LD tagging of SNPs might not be good enough to detect all hair color variants. Finally, for red and black hair color the results may be difficult to interpret due to the lack of power.

When adding the three Dutch PCs as covariates in the model, the estimates of the hair color liability explained by the known hair color loci do not change much (Table 4). However, the estimates of the total autosomal variation, as well as the top chromosomes all drop to almost 0 for blond and brown hair (and therefore light *versus* dark). Although standard errors do not show significant differences between the estimates, it indicates there are still unknown variants that determine blond and brown hair color, which are captured by the PCs, or there are variants that determine traits related to hair color (population stratification).

### 3.4. GWA Analysis

In total, genotype and hair color data were available for 7091 subjects from the NTR. We performed five case-control GWAS with logistic regression for all SNPs including age, sex, the three Dutch PCs and genotype platform as covariates. Familial structure was taken into account in the analysis using Plink and selecting a single monozygotic twin. The resulting Q–Q and Manhattan plots for all colors and light *versus* dark hair color are shown in Figures S1–S10. As shown with GCTA and with the twin heritability modeling, the PC’s are significantly related to hair color in the Netherlands along the three Dutch major axes of genetic variation (Figures S11 and S12). Using the PCs, we corrected for this population stratification, with post correction GWAS λs ranging between 1.004 and 1.027. However, as a consequence, we have likely also reduced the significance of SNPs, which are truly associated with hair color.

#### 3.4.1. Known Hair Color Variants in Relation to the GWAS Results

Table 5 and Table 6 display the gene variants for hair color, which are known from previous studies and our most significant SNPs within these genes. The two genes *HERC2* (15q11.2-13), along with neighboring gene *OCA2*, are known as the most essential genes for determining human pigmentation traits including eye, hair and skin color [15,32]. These genes also show strong signals on chromosome 15 in our study, with SNPs rs7495174 and rs79097182 for blond, brown and light hair color.

The solute carrier (SLC) gene family group is a large family gene group that consists of 458 genes in 52 families. Three loci have been found to be associated with human pigmentation: *SLC24A5*, *SLC45A2* and *SLC24A4* gene. Interactions between *HERC2* and *SLC24A4* play a role in determining blue eye color, but also light hair color and less tanning ability [32,33]. *SLC24A4* (14q32.12) encodes the sodium/potassium/calcium exchanger 4 protein (*NCKX4*). Alternative splicing of this gene results in multiple transcript variants. Variants in *SLC24A4* have been previously associated with eye and hair color, skin sensitivity to sun and coetaneous malignant melanoma [34]. We confirmed the associations for hair color. Within *SLC24A4*, rs8014907 was significantly associated with all hair colors, except red. The *SLC45A2* gene, which is in the same family as *SLC24A4*, encodes a transporter protein that mediates melanin synthesis. The protein of *SLC45A2* is expressed in a high percentage of melanoma cell lines. Mutations in this gene are a cause of oculocutaneous albinism type 4, and polymorphisms in this gene are associated with variations in skin and hair color [35,36]. Multiple transcript variants encoding different isoforms have been found for this gene. Our results do show a *p*-value = 9.1 × 10^−5^ for the gene. *SLC24A5* was found to be involved in skin pigmentation in European populations [33]. A 4-bp insertion (c.569_572insATTA rs1426654) in the *SLC24A5* gene, causing a frame shift and premature termination, was identified in a man with Indian ancestry [37]. Homozygosity of this insertion results in extreme hypopigmentation and pinkish-white skin, with dark brown hair and a brown iris [35]. However, we have not found any significant hits in this locus. For *SLC24A5* gene the lowest *p*-value is 0.7 and the question is whether we have people with this particular insertion present within our Dutch population.

*MC1R* (16q24.3) is an intron-less gene of the size less than 1 kb. Non-synonymous variants in *MC1R* are present in approximately 50% in the European population [38]. Its multiple variants were first found to be associated with human red hair color in 1995 [39]. A subsequent study found these variants to have the same effect on pigmentation at increased frequency with increasing latitude in humans [40,41]. We replicate this association in our study, as *MC1R* is associated with multiple hair colors, except blond.

*KITLG* (12q22) is known to regulate the number of melanocytes during development, melanin distribution and hyper/hypo pigmentation. Sequence variation is thought to affect expression of *KITLG* (184,745), which results in the blond hair color. In European populations rs12821256 T/C SNP is found to be associated with blond hair color [42,43], and this SNP explains 3%–6% of the variance [10]. Our study confirmed this result, rs12821256 showed a significant associations with blond, brown and light *versus* dark hair color. Recently, a functional study showed that this SNP alters a transcription factor binding site for lymphoid enhancer-binding factor 1 (LEF1), reducing *LEF1* responsiveness and enhancer activity in cultured human keratinocytes [44].

IRF4 (6p25.3) is associated with hair color and skin pigmentation [42]. There is a strong association of the A allele of a single-nucleotide polymorphism (SNP) on chromosome 6p25.3, rs1540771, with the presence of freckles in Icelandic and Dutch population samples (discovery OR = 1.40, *p*-value = 3.7 × 10^−18^) [45]. In our study, the most significant SNP rs62389424 is a bit further away (34 kb) and is associated with all hair colors except red (*p* < 1.3 × 10^−5^).

**Table 3:** The explained genetic variance of the hair color liability scale for autosomal common SNPs in GCTA.

Phenotypes	N Cases	N controls	Proportion of Genetic Variance Explained by All Common SNPs (SE)	*p*	Top Chromosome	Proportion of Genetic Variance Explained by Top Chromosome (SE)	*p*	Proportion of Genetic Variance Explained by Known Associated SNPs (SE)	*p*
Blond	1547	1793	0.165 (0.081)	1.1E−02	15	0.014 (0.017)	1.8E−01	0.058 (0.022)	5.75E−40
Brown	1946	1394	0.095 (0.079)	9.3E−02	15	0.011 (0.016)	2.5E−01	0.059 (0.022)	7.88E−39
Red	87	3253	0.246 (0.087)	1.9E−03	16	0.163 (0.025)	3.2E−14	0.069 (0.026)	2.25E−55
Black	66	3274	<0.001 (0.083)	5.0E−01	6	0.031 (0.228)	7.7E−02	0.005 (0.003)	1.2E−02
Light *versus* dark	1890	1450	0.140 (0.080)	2.7E−02	15	0.014 (0.017)	2.0E−01	0.069 (0.026)	6.50E−46

SE: standard error. The given proportion of phenotypic variance explained by SNPs V(G)/V(P) without using a prevalence liability scale transformation.

**Table 4:** The explained genetic variance of the hair color liability scale for autosomal common SNPs in GCTA including the three Dutch PCs as covariates.

Phenotypes	N Cases	N Controls	Proportion of Genetic Variance Explained by All Common SNPs (SE)	*p*	Top Chromosome	Proportion of Genetic Variance Explained by Top Chromosome (SE)	*p*	Proportion of Genetic Variance Explained by Known Associated SNPs (SE)	*p*
Blond	1547	1793	<0.001 (0.084)	0.5	15	0.001 (0.015)	0.48	0.054 (0.024)	2.81E−37
Brown	1946	1394	<0.001 (0.082)	0.5	15	<0.001 (0.016)	0.5	0.054 (0.021)	6.95E−39
Red	87	3253	0.165 (0.025)	1.39E−03	16	0.255 (0.083)	2.36E−14	0.053 (0.020)	1.65E−55
Black	66	3274	<0.001 (0.084)	0.5	6	0.027 (0.228)	0.10	0.005 (0.004)	2.4E−02
Light *versus* dark	1890	1450	<0.001 (0.084)	0.5	15	<0.001 (0.016)	0.5	0.065(0.024)	1.53E−43

SE: standard error. The given proportion of phenotypic variance explained by SNPs V(G)/V(P) without using a prevalence liability scale transformation.

**Table 5:** SNP associations within our study for blond and brown hair against known hair color loci.

Locus	Chromosome Location	Most Significant SNP	MAF	OR Blond	SE Blond	*p* Blond	OR Brown	SE Brown	P Brown
SLC45A2	5p13.2	rs16891982 *	0.050	0.5036	0.1752	9.1E−05	1.4506	0.1656	0.02472
IRF4	6p25.3	rs1540771	0.489	1.0253	0.1978	0.003	0.9027	0.0396	9.7E−03
IRF4	6p25.3	rs62389424 *	0.087	2.4846	0.1188	1.3E−13	0.5551	0.1081	5.2E−08
TYRP1	9p23	rs1408799 *	0.291	0.8522	0.0484	0.001	1.1846	0.0468	2.9E−03
TPCN2	11q13.3	rs72930659	0.109	0.6073	0.0657	3.2E−14	1.5544	0.0649	1.1E−11
KITLG	12q21.33	rs12821256	0.128	0.6715	0.0616	9.8E−11	1.4366	0.0596	1.2E−09
SLC24A4	14q32	rs8014907	0.178	1.4317	0.0554	3.0E−10	0.7262	0.0529	3.1E−09
SLC24A5	15q21.1	rs1834640	0.110	1.0163	0.1323	0.903	0.9858	0.1294	0.9121
HERC2	15q13	rs79097182	0.038	2.9822	0.1426	1.8E−14	0.4827	0.1235	3.7E−09
OCA2	15q13.1	rs7495174 *	0.014	3.5485	0.2577	8.9E−07	0.3544	0.2168	1.7E−06
MC1R	16q24	rs2353688	0.028	1.8309	0.1432	2.4E−05	0.6028	0.1316	1.2E−04
MC1R	16q24	rs146972365	0.053	0.9765	0.0945	0.802	1.7771	0.0922	4.5E−10
MC1R	16q24	rs8063160	0.065	0.9119	0.0872	0.290	1.7837	0.0847	8.6E−12
MC1R	16q24	rs117322171	0.014	0.9469	0.1795	0.761	0.9039	0.1789	0.572
ASIP	20q11.22	rs1015362	0.273	1.0448	0.0464	0.344	0.9559	0.0452	0.3181
ASIP	20q11.22	rs4911414	0.347	0.9922	0.0428	0.855	1.0148	0.0417	0.724

* These SNPs failed the imposed quality control in our sample: rs62389424 (HWE *p* = 2.6E-21), rs7495174 (HWE *p* = 1.27203E-12), rs16891982 (HWE *p* = 1.05107E-05), rs1408799 (MAF difference with imputation reference set >0.15).

**Table 6:** SNP associations within our study for light *versus* dark, red and black hair against known hair color loci (same loci as Table 5).

Most Significant SNP	OR Red	SE Red	*p* Red	OR Black	SE Black	*p* Black	OR Light *versus* Dark	SE Light *versus* Dark	*p* Light *versus* Dark
rs16891982 *	0.8443	0.6410	0.792	4.9177	0.3258	1.0E−06	1.9867	0.1764	1.0E−04
rs1540771	0.6853	0.367	0.010	0.8300	0.1112	0.104	0.6822	0.307	0.002
rs62389424 *	0.6897	0.3084	0.228	0.3985	0.2107	1.3E−05	0.4424	0.1171	3.4E−12
rs1408799 *	0.9043	0.1467	0.493	0.9806	0.1355	0.885	1.1823	0.0478	4.6E−04
rs72930659	1.2327	0.2211	0.344	1.3688	0.1968	0.1106	1.6087	0.0653	3.4E−13
rs12821256	1.0088	0.1951	0.964	1.3023	0.1895	0.1634	1.4829	0.0612	1.2E−10
rs8014907	1.0271	0.1788	0.709	0.8070	0.1453	0.1400	0.7028	0.0547	2.6E−10
rs1834640	1.1650	0.4093	0.831	0.8708	0.3677	0.7067	0.9691	0.1315	0.811
rs79097182	0.5954	0.3135	0.098	0.4352	0.2254	2.2E−04	0.3900	0.1386	5.2E−14
rs7495174 *	0.6939	0.5975	1.7E−06	0.9691	0.3839	0.9349	0.3216	0.2443	3.4E−06
rs2353688	0.5149	0.3862	0.086	1.4452	0.4024	0.3601	0.6148	0.1348	3.1E−04
rs146972365	0.0720	0.1674	1.1E−55	1.8403	0.3244	0.6007	1.8751	0.0924	1.0E−11
rs8063160	0.0770	0.1728	7.8E−50	1.9278	0.3041	0.3090	1.8891	0.0845	5.2E−14
rs117322171	1.7219	0.6122	0.375	452.3873	1.0414	4.3E−09	1.0119	0.1771	0.947
rs1015362	0.9583	0.1500	0.776	1.0513	0.1282	0.6967	0.9619	0.0464	0.402
rs4911414	0.7644	0.1371	0.725	1.1988	0.1200	0.1309	1.0366	0.0426	0.399

* These SNPs failed the imposed quality control in our sample: rs62389424 (HWE *p* = 2.6E-21), rs7495174 (HWE *p* = 1.27203E-12), rs16891982 (HWE *p* = 1.05107E-05), rs1408799 (MAF difference with imputation reference set >0.15).

Genetic variants in 3-prime-untranslated region of the ASIP result in skin/hair/eye pigmentation variation. ‘The *ASIP* haplotype’, rs1015362G and rs4911414T was shown to be associated with red hair color, freckling, and skin sensitivity to sun, in addition to burning and freckling that reached genome wide significance (max odds ratio = 1.60, *p*-value = 3.9 × 10^−9^) in the Icelandic and Dutch populations [45]. In our study, however, we did not find any significant results for the *ASIP* locus. Minimum *p*-values for these genes were above 0.35 for the hair colors. Note that red hair color in our sample is not so frequent, so this could be related to detection power.

*TPCN2* (11q13.3.) was found to be significantly associated with blond *versus* brown hair color in Icelandic Europeans [42]. In our study SNPs in this locus are significantly associated with blond, brown, black and light *versus* dark hair colors (*p* < 4.7 × 10^−10^).

The variants of *TYRP1* gene are known skin/hair/eye pigmentation variation locus. A suggestive association for blond *versus* brown hair was observed for rs1408799 in Iceland and Dutch populations, and functional data suggest that the *TYRP1* gene encodes a melanosomal enzyme with a role in the eumelanin pathway [45]. The *p*-values in our study are about 10^−4^ implying a potential, but weak association with hair color.

#### 3.4.2. Identification of New Hair Color Variants from the NTR GWAS Results

Within this study, no new SNPs were significantly associated with hair color after conservatively filtering on MAF > 0.01 for blond, brown and light *versus* dark hair color and MAF > 0.05 for red and black hair color. Initially we had some associations for black and red hair color when also filtering on MAF > 0.01. However, with this threshold of filtering the number of cases having the minor allele(s) is extremely small, which leads to inflated statistics. Subsequently, these were not confirmed as positive results as none of the red hair color findings (black hair color unavailable) were replicated by the Decode study, and permutation analyses showed that the findings were also likely under the hypothesis of no association (Table S7).

## 4. Conclusions

Our twin family analysis shows high heritability for hair color (70%–97%). Both additive and non-additive genetic models, as well as positive assortative mating and population stratification should be taken into consideration when conducting genetic studies of these traits in the Dutch population. In the GWA analysis we could confirm previously known associated variants in the *MC1R* and *HERC2*, *TPCN2*, *SLC24A4*, *IRF4* and *KITLG* genes. The GCTA analyses shows that common SNPs in these loci explain about 6% of the hair color liability in our population. In total, between 0% and 25% and, on average, roughly 13% of the hair color liability can be explained by common SNPs genome wide, and therefore new variants, either rare and tagged by the common SNPs or simply not identified yet, are likely still present within the genome. This study also shows the issues of current standard GWAS approaches. The modeling assumptions of GCTA assume additive effects with many genes influencing the trait. However, as made evident, there are non-additive effects, assortative mating, population stratification and the likelihood of involvement of rare gene variants and the question is thus whether estimates of the variance explained by these methods are optimal. To find the missing heritability, an investment in large sample sizes with meta-analysis and methodological innovation to deal with these other-than-additive circumstances, a stratified population and better rare allele detection is needed to improve locus detection, even for a highly heritable trait like hair color.

## Supplementary Files

Supplementary File 1
