# Supplementary material for: Heritability and Genome-Wide Association Studies for Hair Color in a Dutch Twin Family Based Sample
Source: Genes (Basel). 2015 Jul 13;6(3):559–76. doi: 10.3390/genes6030559 (PMC4584317; doi:10.3390/genes6030559)
Supplement: Supplementary File 1 [file genes-06-00559-s001.pdf]

Supplementary Materials

Variance Decomposition including Assortative Mating within the Applied Twin Model

The variance of the liability underlying the phenotype, V_{ph}, is standardized, *i.e.*, $V_{ph} = 1$. The variance is decomposed as follows $V_{ph} = V_A + V_D + V_E$, where, additive genetic (V_A), the dominance genetic (V_D) and the unshared environmental variance components (V_E) sum to one, and two parameters are estimated as free parameters. The broad-sense heritability equals $V_A + V_D$, and the narrow-sense heritability equals V_A. In the notation of Falconer and MacKay (1996) [1], we use r to denote the spousal phenotypic correlation, and m to denote the correlation between the parental breeding values, *i.e.*, $m = r \times V_A$. Given the standardization, we express the expected phenotypic covariances in terms of correlations. These are:

Spousal:	r
Parent-offspring covariance:	$½V_A (1 + r)$
Monozygotic twin correlation:	$V_A + V_D$
Dizygotic twin and full sib correlation:	$½V_A (1 + m) + ¼V_D = ½V_A (1 + r \times V_A) + ¼V_D$

The dizygotic twin, and full sib correlation, follows from $m = r \times V_A$ given the assumption of purely phenotypic assortment [1]. Note that the phenotype spousal correlation (r) may differ when it is estimated directly on the basis of the parental data as compared to the correlation estimated in the full model, as in the latter the dizygotic twin and full sib correlations also play a role.

We also considered an ACE model (in the case of red and black hair colors), where C stands for common environmental influences shared by family members. Letting V_C denote the shared environmental variance, the expected correlations sib correlations are $V_A + V_C$ (MZs) and $½V_A (1 + r \times V_A) + V_C$ for full sibling and dizygotic twins.

Table S1. The sample of family members within the twin-family modeling study (N = 20,142).

Members	N	Age	Blond	Red	Light Brown	Dark Brown	Black
Fathers	1190	67.16 ± 6.52	458	39	227	349	117
Mothers	1584	64.40 ± 7.35	449	48	412	608	67
Twins	15,359	31.25 ± 14.19	6245	759	3840	4147	368
Brothers	370	31.27 ± 13.90	159	10	89	98	14
Sisters	1639	32.25 ± 13.72	772	55	410	384	18

Table S2. Zygosity information for the subsample of twins within the twin modeling study (N = 15,359).

Zygosity	N	Blond	Red	Light Brown	Dark Brown	Black
Monozygotic males	2345	904	101	534	709	97
Dizygotic males	1678	632	75	396	495	80
Monozygotic females	4651	1905	280	1196	1211	59
Dizygotic females	2710	1095	154	753	676	32
Opposite-sex twins	3975	1709	149	961	1056	100

Table S3. Characteristics for subjects in the GWA study (N = 7,091) and the sub-selection of unrelated individuals from these subjects for the GCTA study (N = 3,340).

Member	N	Blond	Red	Light Brown	Dark Brown	Black
Fathers	657	292	14	101	187	63
Mothers	1021	329	22	240	395	35
Twins	4320	1828	115	1103	1205	69
Siblings	882	353	25	246	235	23
Spouses	211	86	5	53	61	6
GCTA Unrelated	3340	1547	87	1019	927	66

Table S4. Results of the variance components model fitting for each hair color. The saturated model is compared to the ADE, ACE (where applicable) and AE models. Also the effects of including age as a covariate, sex limitations (quantitative and qualitative) and assortative mating are examined.

Hair color	Model	P1	P2	−2LL	df	AIC	DLL	Ddf	*p*-Value
Blond	SAT	15	15	23,837.1	20,127	−16,416.9			
	Full ADE	9	7	23,964.98	20,135	−16,105.02	127.88	8	1.74E−24
	ADE m = f	6	5	24,004.14	20,137	−16,271.86	167.04	10	1.14E−30
	Age = 0	8	6	24,135.06	20,136	−16,136.94	297.96	9	7.07E−59
	r = 0	8	6	23,983.35	20,136	−16,288.65	146.25	9	5.27E−27
	AE	7	5	24,123.37	20,137	−16,150.63	286.27	10	1.24E-55
Brown	SAT	15	15	24,974.3	20,127	−15,279.7			
	Full ADE	9	7	25,027.81	20,135	−15,242.19	53.51	8	8.59E−09
	ADE m = f	6	5	25,080.32	20,137	−15,195.68	106.02	10	3.38E−18
	Age = 0	8	6	25,057.71	20,136	−15,214.29	83.42	9	3.37E−14
	r = 0	8	6	25,036.46	20,136	−15,235.54	62.16	9	5.13E−10
	AE	7	5	25,360.84	20,137	−14,917.16	386.54	10	6.88E−77
Red ACE	SAT	15	15	6250.44	20,127	−33,950.93			
	Full ACE	9	7	6336.7	20,135	−33,938.53	86.26	8	2.66E−15
	ACE m = f	6	5	6344.4	20,137	−33,939.55	93.95	10	8.79E−16
	Age = 0	8	6	6382.29	20,136	−33,895.88	131.85	9	4.94E−24
	r = 0	8	6	6419.43	20,136	−33,918.18	169.99	9	6.20E−32
	AE	7	5	6373.81	20,137	−33,915.86	123.37	10	1.05E−21
Black ACE	SAT	15	15	4597.64	20,127	−35,654.36			
	Full ACE	9	7	4618.18	30,135	−35,670.79	20.54	8	8.48E-03
	ACE m = f	6	5	4620.21	20,137	−35,665.82	22.57	10	0.012
	Age = 0	8	6	4795.28	20,136	−35,510.64	197.64	9	1.03E-37
	r = 0	8	6	4621.38	20,136	−35,645.17	23.74	9	4.7E-03
	AE	7	5	4621.3	20,139	−35,647.38	23.66	10	8.56E-03

Table S4. ***Cont.***

Hair color	Model	P1	P2	−2LL	df	AIC	DLL	Ddf	*p*-Value
Light *versus* dark	SAT	15	15	24,356.28	20,127	−15,897.72			
	Full ADE	9	7	24,439.25	20,135	−15,830.75	82.97	8	1.23E-14
	ADE m = f	6	5	24,472.93	20,137	−15,803.07	116.65	10	2.42E-20
	Age = 0	8	6	24,533.94	20,136	−15,738.06	177.66	9	1.56E-33
	r = 0	8	6	24,456.93	20,136	−15,815.07	100.65	9	1.16E-17
	AE	7	5	24,578.55	20,137	−15,695.45	222.27	10	3.58E-42

P1: the number of parameters, P2: the number of independent parameters (taking into account the standardization of the phenotypic liability), −2LL: −2loglikelihood, df: total degrees of freedom, AIC: Akaike Information Criterion, DLL: difference in −2loglikelihood, Ddf: difference in degrees of freedom, compared to saturated model. SAT: saturated model, Full ADE: ADE model (including assortative mating (r), age as a covariate, and sex limitation), Full ACE: ACE model (including assortative mating (r), age as a covariate, and sex limitation), ADE/ACE m = f: ADE/ACE model without quantitative sex limitations, Age = 0: ADE/ACE model without age as covariate, r = 0: ADE/ACE model without assortative mating effect, AE: AE model without dominance genetic effects or common environment effects.

Table S5. Specific tests of including age as a covariate, sex differences and assortative mating as compared to the full ADE/ACE model for each hair color.

Hair color	Model Comparison	DLL	Ddf	*p*-value
Blond	ADE m = f	39.16	2	3.14E−09
	Age = 0	170.08	1	7.11E−39
	r = 0	18.37	1	1.82E−05
	AE	158.39	2	4.04E−35
Brown	ADE m = f	52.51	2	4.28E−13
	Age = 0	29.91	1	4.53E−08
	r = 0	8.65	1	3.27E−03
	AE	333.03	2	4.82E−73
Red ACE	ACE m = f	7.7	2	0.05
	Age = 0	45.59	1	1.46E−11
	r = 0	82.73	1	9.41E−20
	AE	37.11	2	8.74E−09
Black ACE	ACE m = f	2.04	2	0.56
	Age = 0	177.1	1	2.08E−40
	r = 0	3.2	1	0.07
	AE	3.12	2	0.21
Light v*ersus* dark	ADE m = f	33.65	2	4.93E−08
	Age = 0	94.69	1	2.23E−22
	r = 0	17.68	1	2.61E−05
	AE	139.3	2	5.64E−31

DLL: Difference in −2 log likelihood, Ddf: difference in degrees freedom.

Table S6. Estimates from ADE model for hair color with—and without PC correction (compared with saturated model, N = 5777 individuals having genotype data and are fitting the twin modeling family characteristics).

Color	Model	Age	PC1	PC2	PC3	RSpouse/r	Am	Dm	Em	Af	Df	Ef	−2LL	P2	DLL	Ddf	*p*
Blond	M1	0.084	−11.33	−0.44	−1.81	0.281							6801.08	18			
	M2	0.083	−11.11	−0.43	−1.94	0.259	0.45	0.50	0.05	0.71	0.25	0.04	6830.85	10	29.77	8	2.32E−04
	M3	0.068				0.272	0.50	0.45	0.05	0.71	0.25	0.04	6876.9	7	75.83	3	2.41E−16
Brown	M1	−0.045	9.90	1.34	0.04	0.334							7049.92	18			
	M2	−0.048	9.44	1.11	0.06	0.225	0.23	0.71	0.06	0.65	0.30	0.05	7074.68	10	24.76	8	1.71E−03
	M3	−0.037				0.234	0.25	0.69	0.06	0.65	0.31	0.04	7110.94	7	61.02	3	3.56E−13
Light	M1	−0.097	10.82	1.20	0.80	0.270							6868.74	18			
versus	M2	−0.098	10.56	1.14	0.83	0.248	0.46	0.49	0.05	0.74	0.22	0.04	6895.23	10	26.49	8	8.66E−04
dark	M3	−0.085				0.259	0.48	0.47	0.05	0.73	0.22	0.05	6937.31	7	68.57	3	8.64E−10

M1: saturated model with age and 3PC's as covariates. M2: genetic models (ADE model for blond, brown and light *versus* dark hair), M3: genetics models without 3PC's as covariates compared with full genetic model. Age, PC1, PC2, PC3: Beta coefficient of regression on age, and first to third principal component. rSpouse/r: tetrachoric correlation of spouse in M1, and assortative mating coefficient in M2 and M3, Am and Af = additive variance, Dm and Df = non-additive variance, Em and Ef = unique environment variance plus measurement error for males (m) and females (f), −2LL: −2loglikelihood, P2: the number of independent parameters (taking into account the standardization of the phenotypic liability), DLL: the difference in −2loglikelihood, Ddf: difference in degrees of freedom, compared to saturated model, P: *p*-Value of chi square test comparing different sub-models.

Table S7. Summary of results of standard association tests of rare SNPs (MAF 0.01–0.05) within the NTR discovery sample. Permutation tests indicate non-significance of the associations. Replication in the Decode cohort also indicate non-significance.

CHR	BP	Most Significant SNP	Hair Color	GENE	*p*-Value	MAF	Odds Ratio	SE	Permutation (N = 10,000) Empirical P	Decode Replication *p*-Value
1	199,471,603	rs74230273	black		1.90E−14	0.015	790	0.87	0.1487	NA
2	172,770,696	rs7563076	black		2.10E−10	0.013	>1000	1.11	0.2084	NA
3	5,984,546	rs149685327	black		1.40E−15	0.013	240	0.69	0.3316	NA
5	172,347,359	rs17074690	black		1.20E−08	0.024	61	0.72	0.0115	NA
6	166,860,270	rs2281057	black	RPS6KA2	1.40E−43	0.028	0.93	0.09	0.2237	NA
6	166,446,782	rs191122540	black	RPS6KA2	1.40E−43	0.028	>1000	13.35	0.3316	NA
7	122,530,292	rs183059797	black		4.50E−08	0.01	0.06	0.5	0.2754	NA
8	76,579,713	rs10993446	black		5.60E−09	0.012	160	0.87	0.2514	NA
9	97,759,920	rs12289701	black		9.00E−16	0.013	>1000	2.19	0.0106	NA
11	44,921,416	rs700019	red	TSPAN18	8.50E−17	0.015	320	0.69	0.0881	NA
1	216,416,300	rs4972217	red	USH2A	8.90E−22	0.012	>1000	1.83	0.3061	0.5765
2	88,842,075	rs7691567	red		5.70E−12	0.011	>1000	9.32	0.2213	0.7509
4	35,892,635	rs28407071	red		1.00E−27	0.015	>1000	1.61	0.0543	NA
7	111,917,427	rs16904010	red	ZNF277	8.80E−22	0.013	400	0.64	0.1537	NA
8	91,163,167	rs10506044	red		1.80E−16	0.014	1000	0.84	0.0514	0.7326
12	29,007,640	rs189573811	red	PDE3A	1.00E−19	0.019	>1000	10.34	0.0514	0.7665
18	6,083,752	rs7238024	red	MC4R	1.20E−22	0.016	>1000	1.8	0.0432	NA
19	4,513,559	rs4807597	red	PLIN4	2.40E−15	0.007	>1000	0.88	0.2391	0.4494
21	20,177,103	rs2825137	red		3.00E−19	0.012	>1000	1.97	0.0487	NA
22	26,842,941	rs112390186	red	HPS4	5.90E−12	0.015	64	0.61	0.3800	0.9565

Chr: chromosome, MAF: minor allele frequency, SE: standard error of odds ratio.

Some of these genes are biologically interesting candidates. MC4R belongs to the melanocortin receptor family, involved in a wide range of physiological functions, including pigmentation. Mutations in HPS4 result in subtype 4 of Hermansky-Pudlak syndrome, a form of albinism as reported in Hutten *et al.* (2008) [2]. USH2A (Usher syndrome 2A) is previously found to be related to retinitis pigmentosa (Seyedahmadi *et al.* 2004) [3].

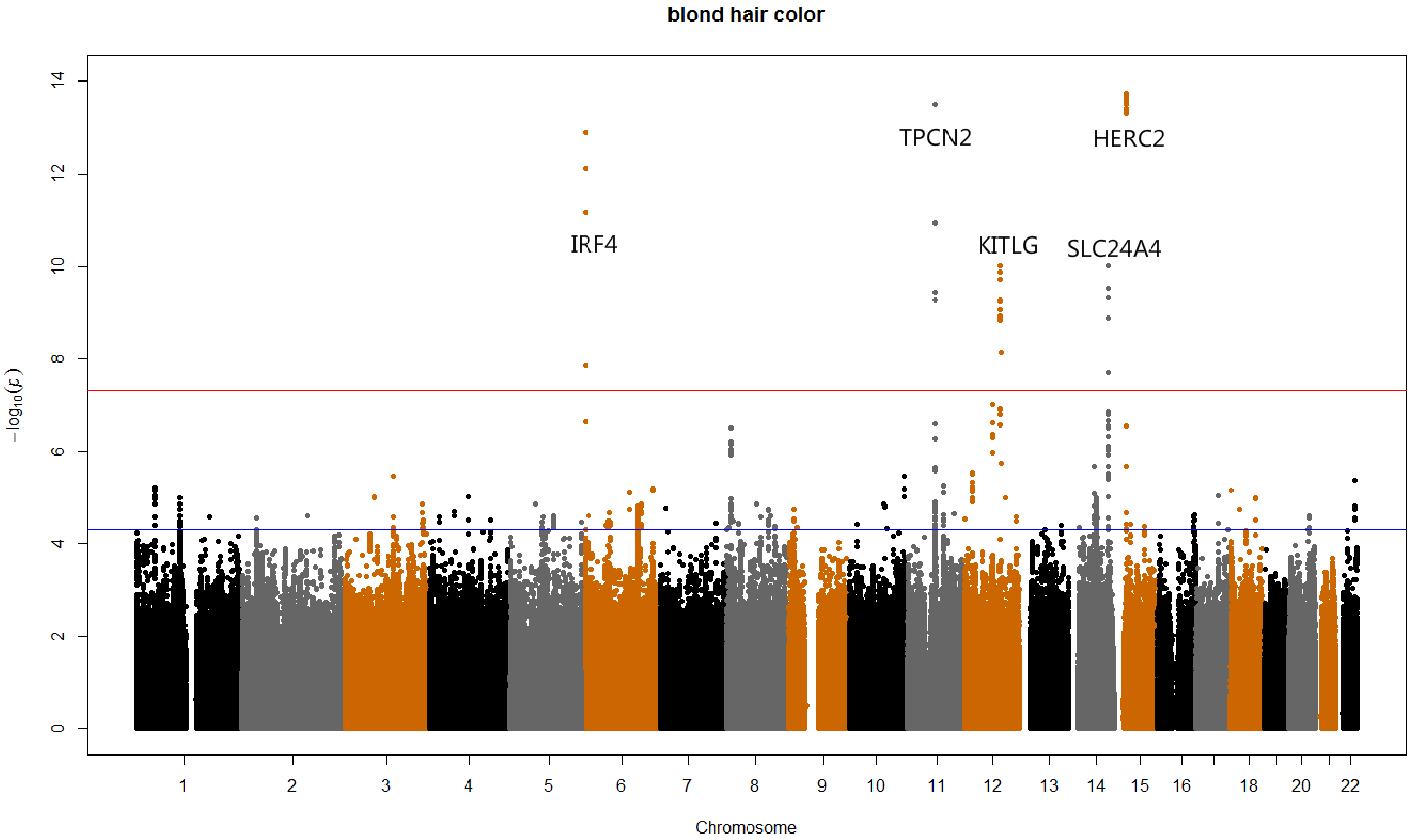

Figure S1. Manhattan plot for blond hair color (MAF > 0.01).

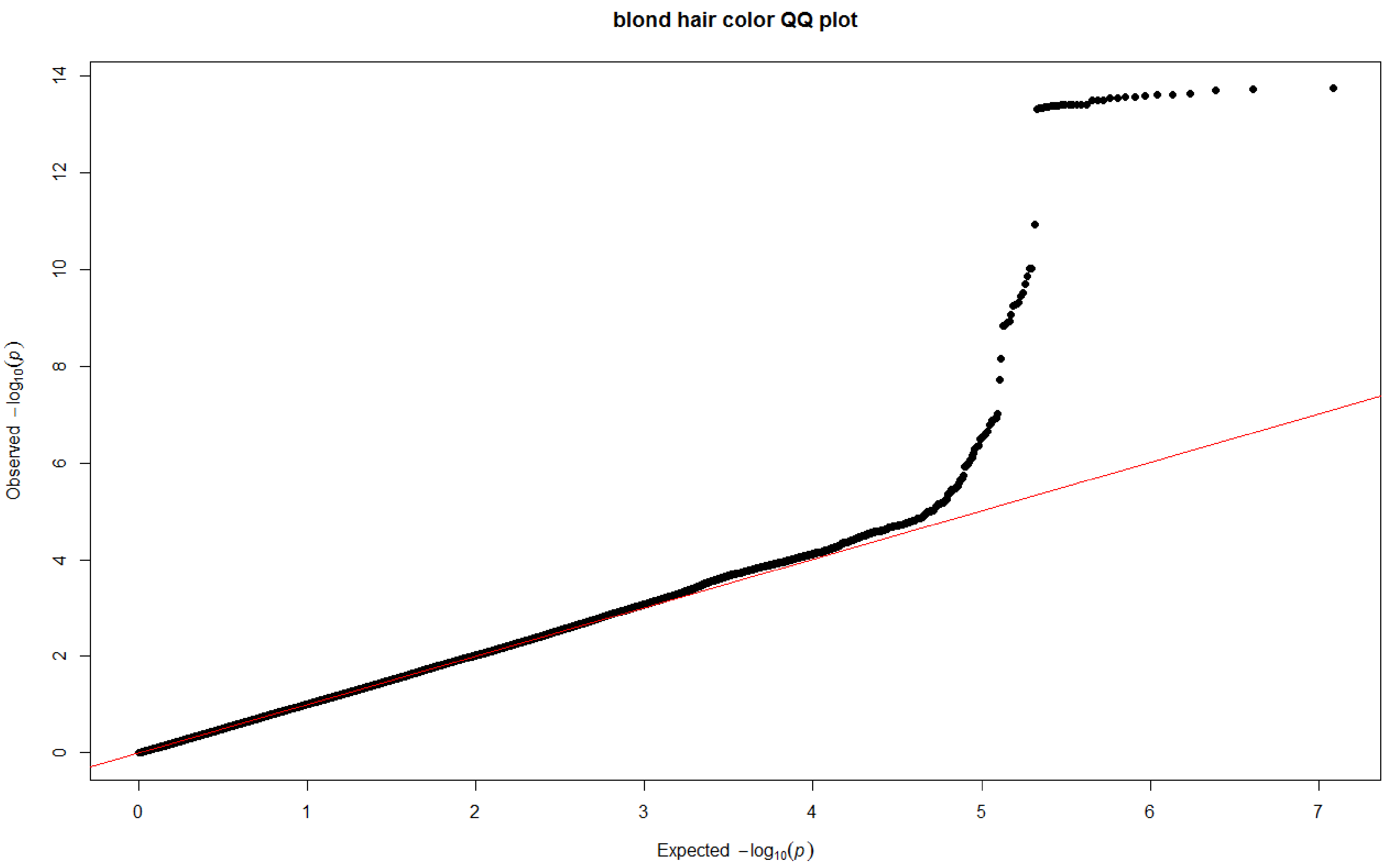

Figure S2. QQ plot for blond hair color (MAF > 0.01). (λ = 1.004673).

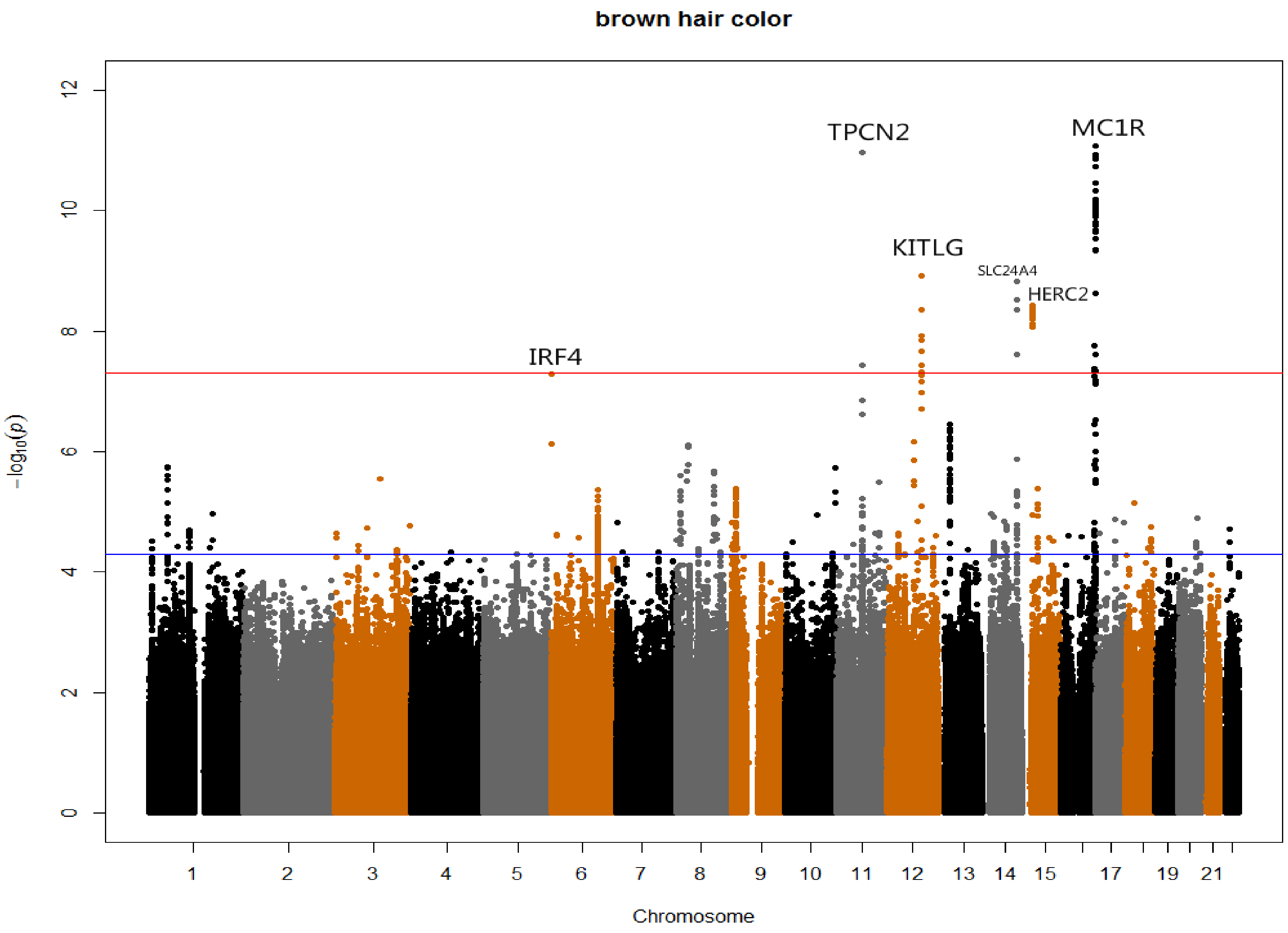

Figure S3. Manhattan plot for brown hair color (MAF > 0.01).

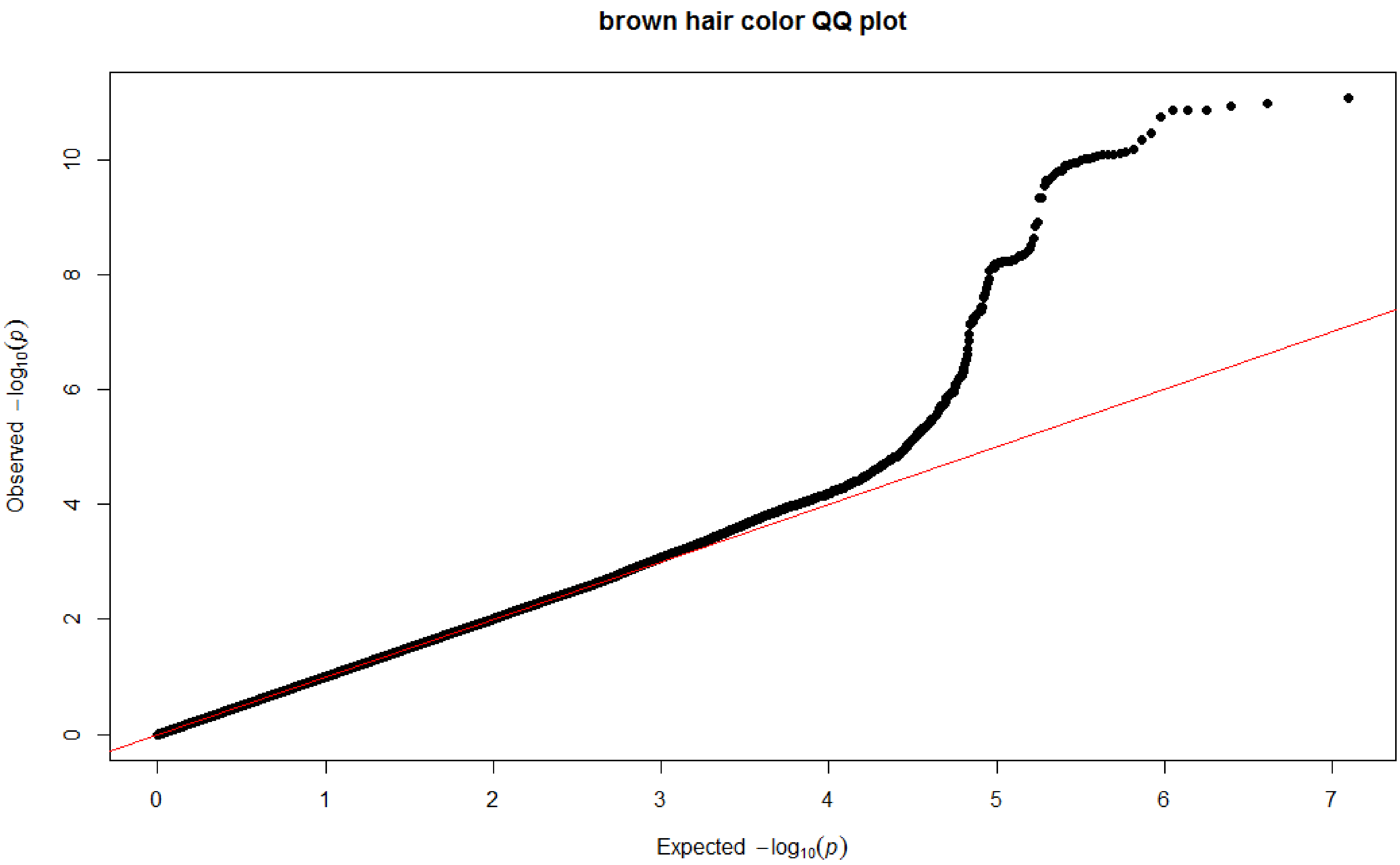

Figure S4. QQ plot for brown hair color (MAF > 0.01). (λ = 1.003738).

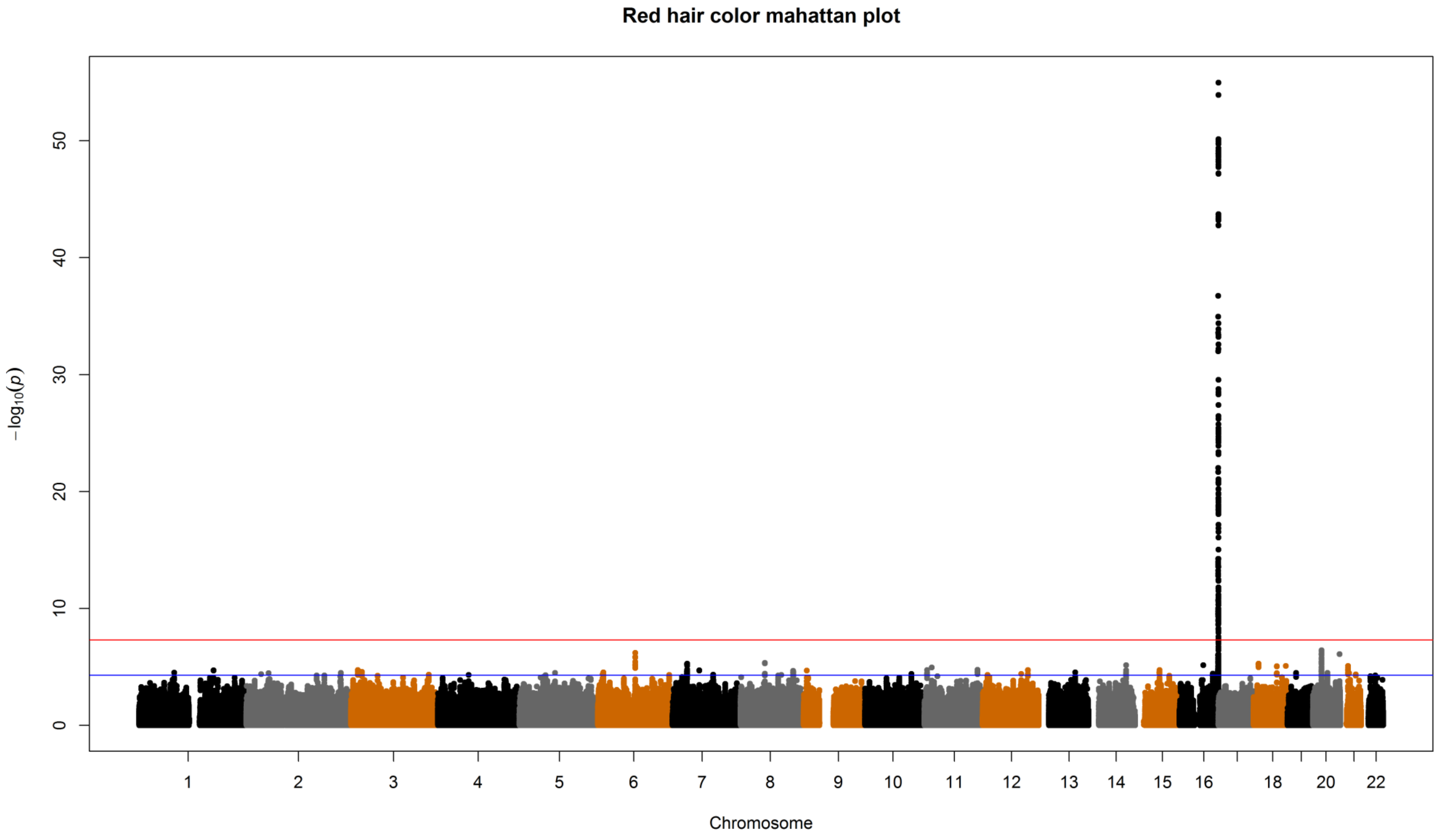

Figure S5. Manhattan plot for red hair color (MAF > 0.05).

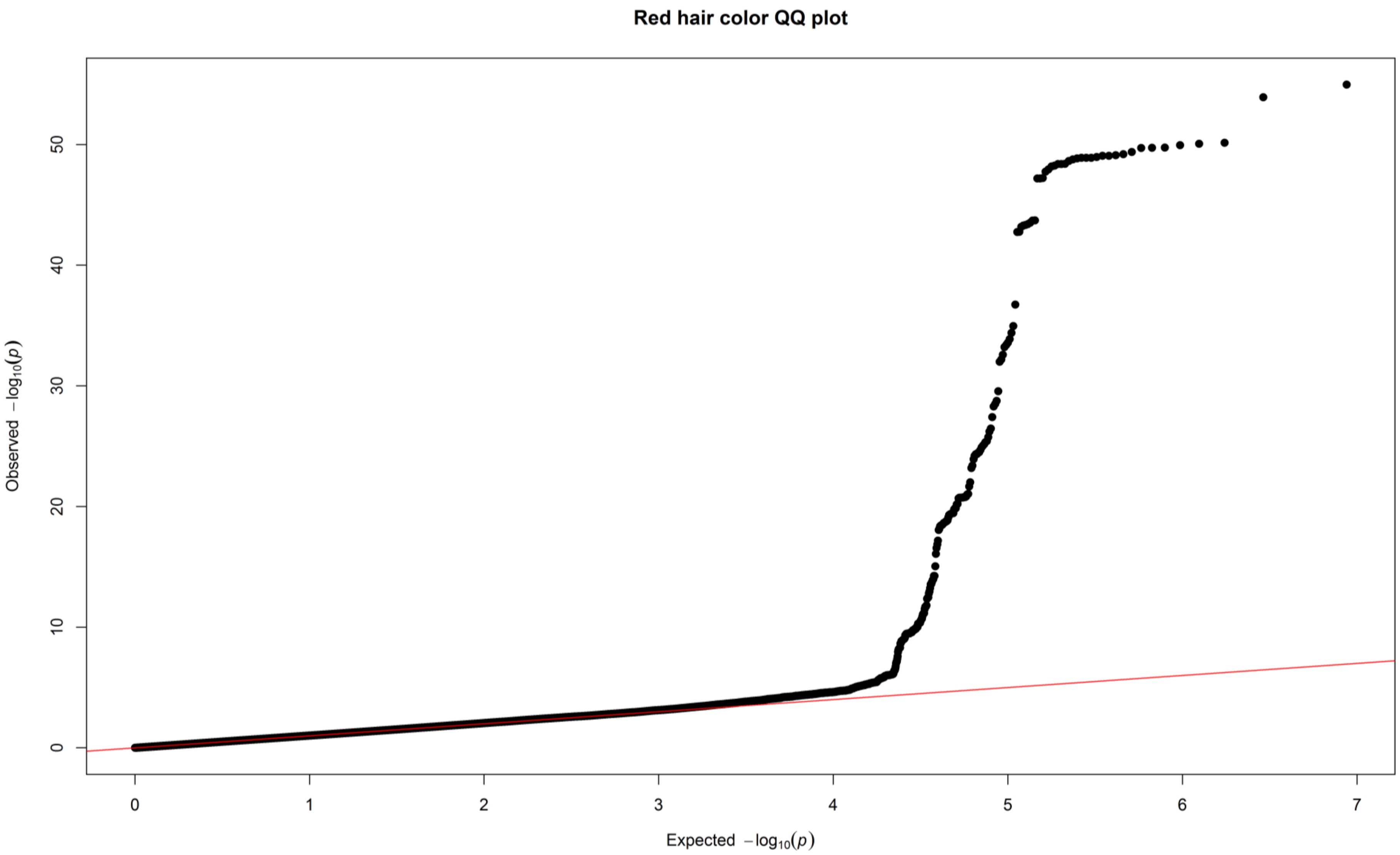

Figure S6. QQ plot for red hair color (MAF > 0.05). (λ = 1.021156).

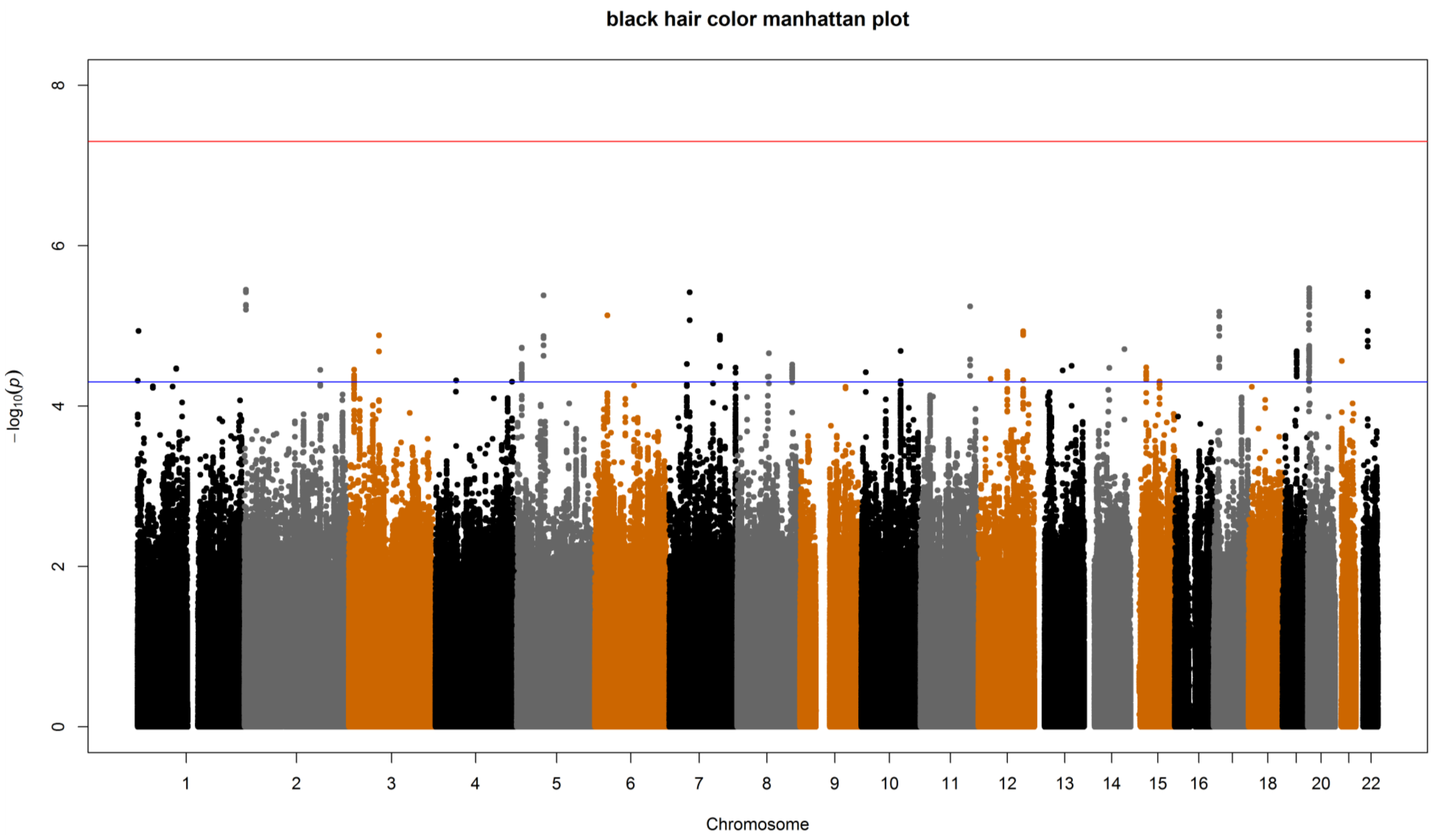

Figure S7. Manhattan plot for black hair color (MAF > 0.05).

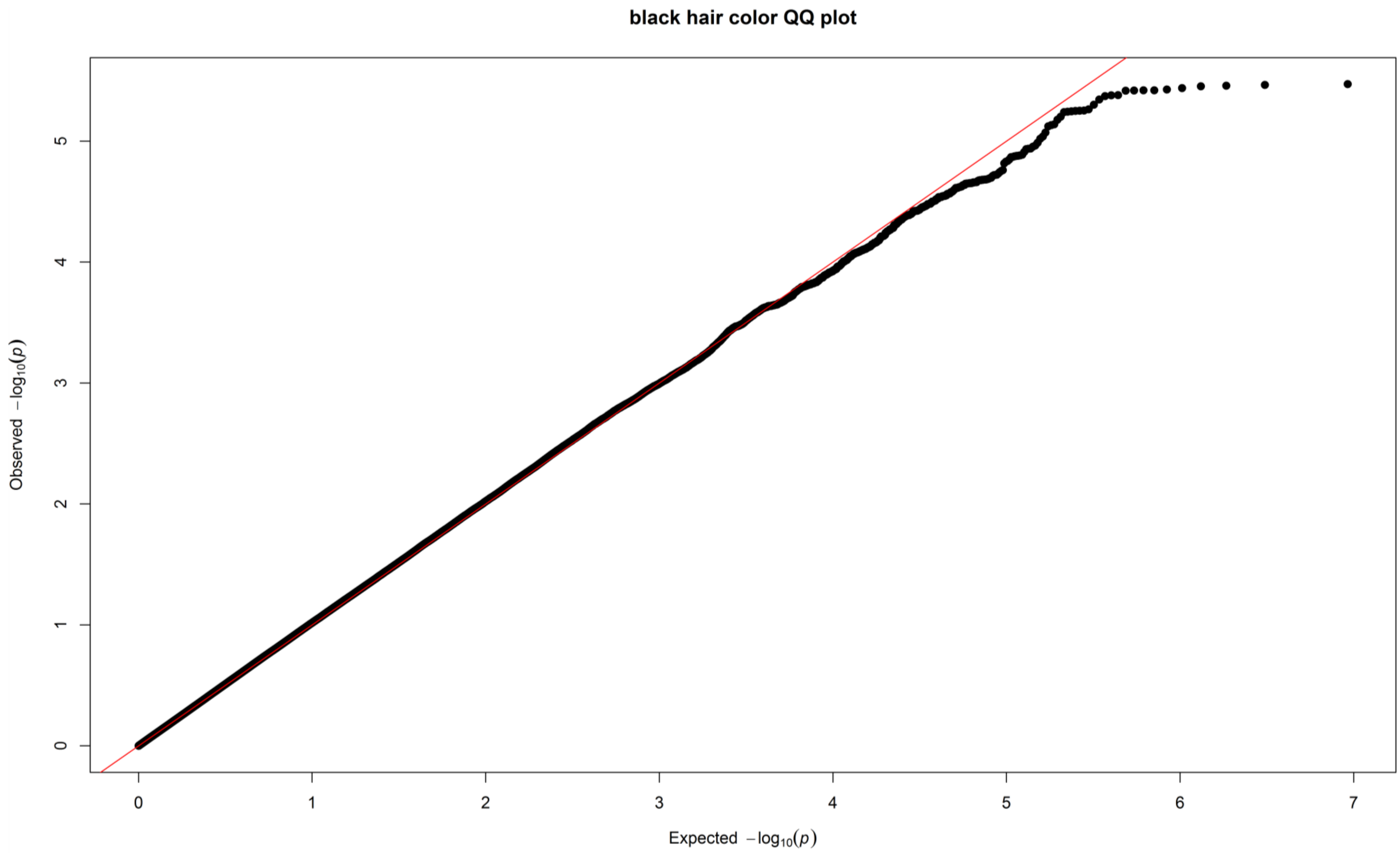

Figure S8. QQ plot for black hair color (MAF > 0.05). (λ = 1.027328).

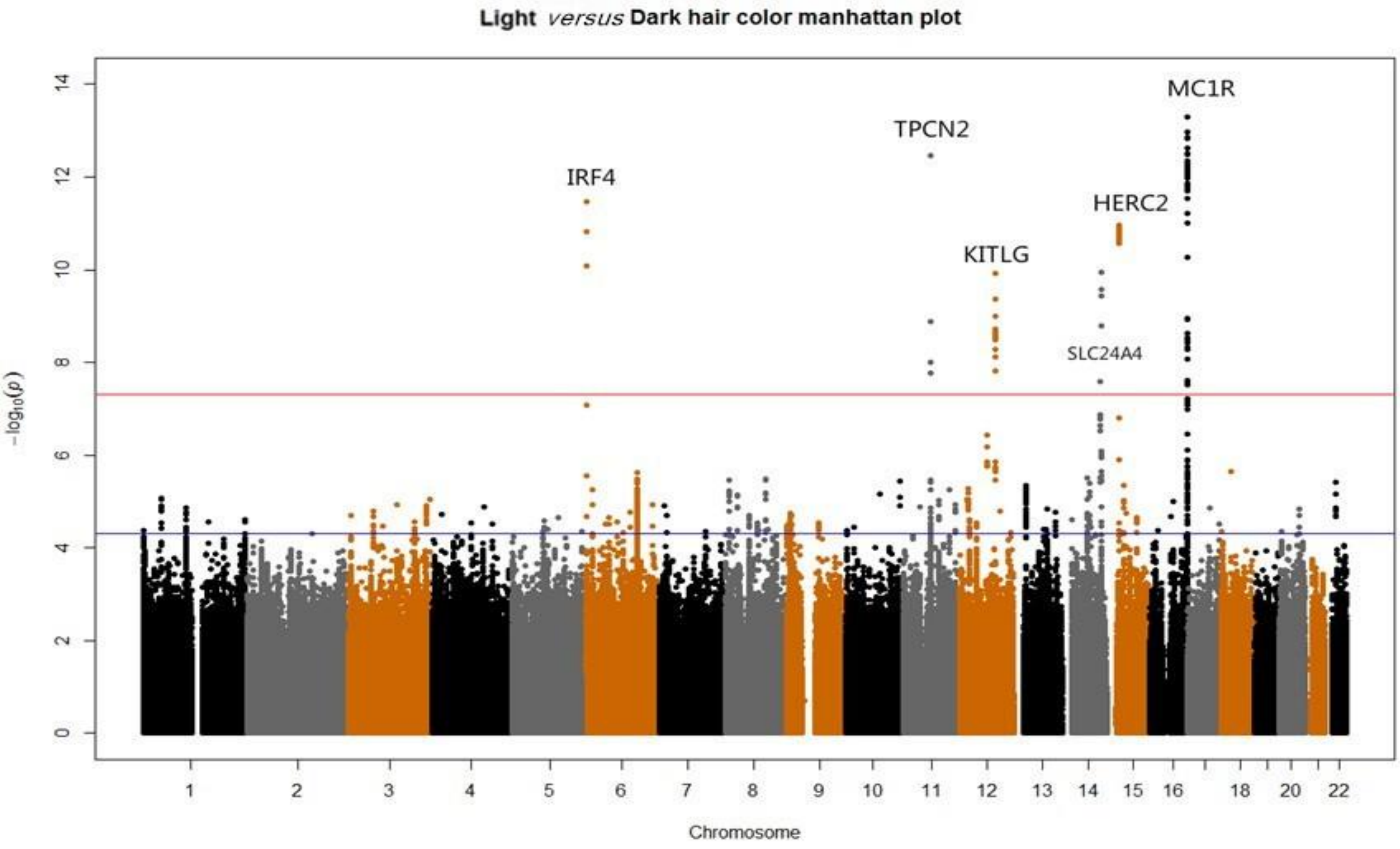

Figure S9. Manhattan plot for light *versus* dark hair color (MAF > 0.01).

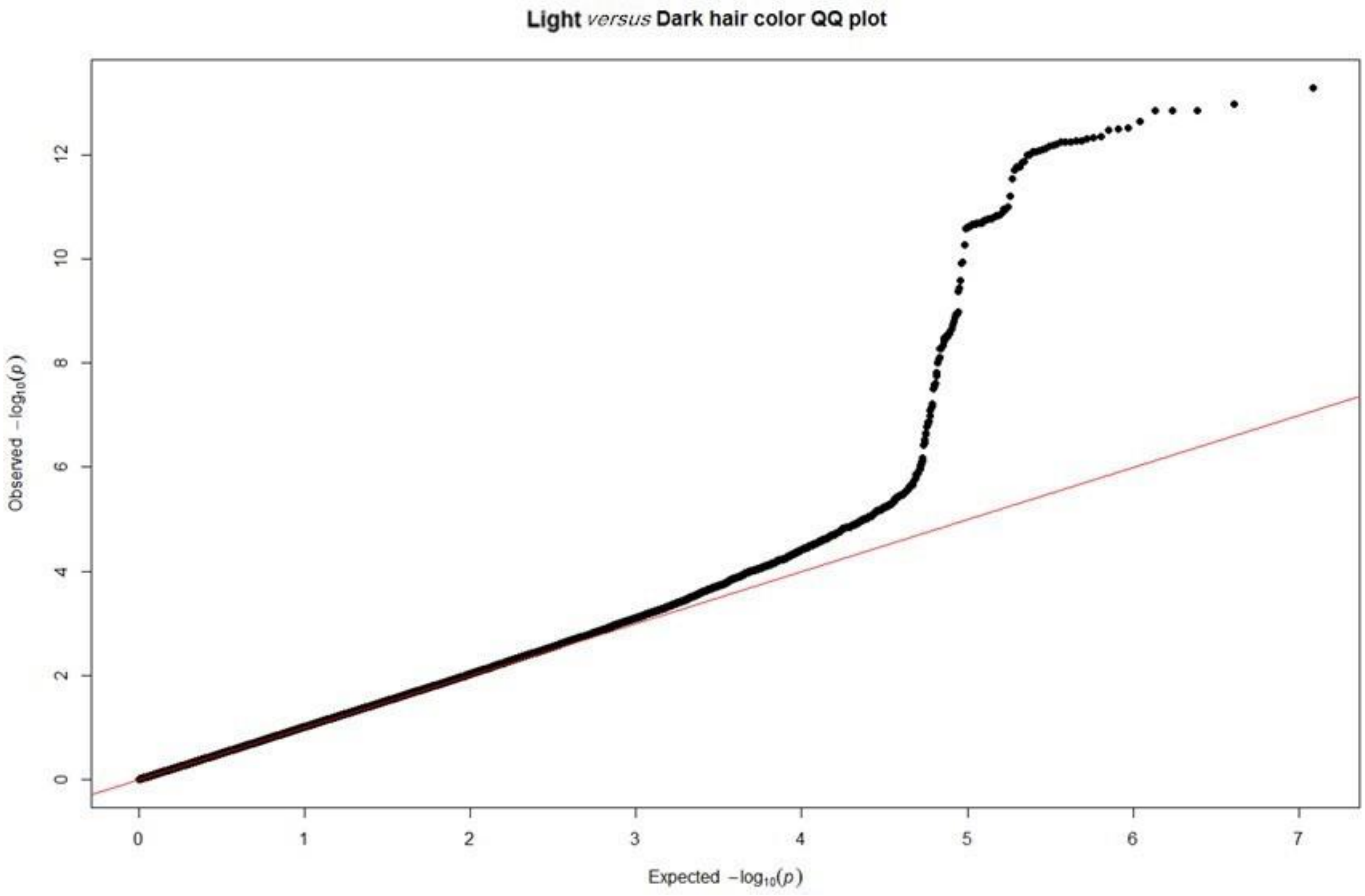

Figure S10. QQ plot for light *versus* dark hair color (MAF > 0.01). (λ =1.006079).

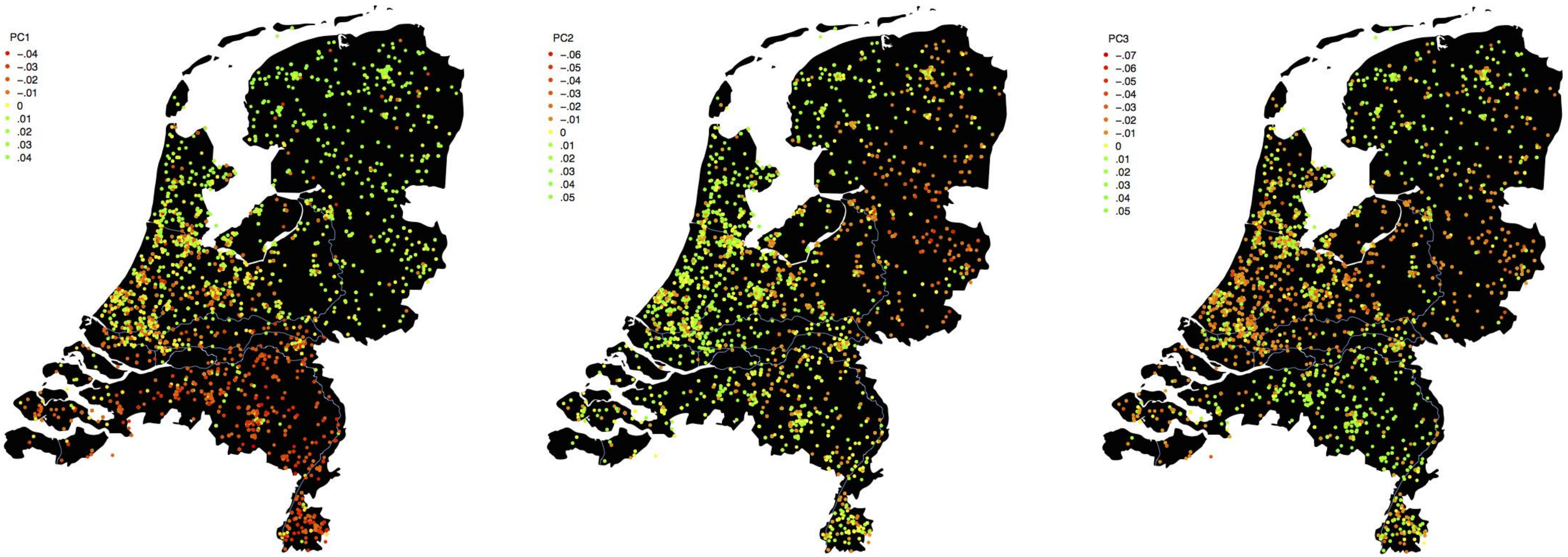

Figure S11. The three genomic PCs and their correlation with geography.

These figures show a map of the Netherlands, where the relation between geography and the genetic principal components 1 to 3 is plotted. The colors of the points indicate the mean value per postal code of PC1, PC2 and PC3 respectively. The plot is based on 7091 Dutch individuals with current address postal code information, hair color phenotype and genetic information [4].

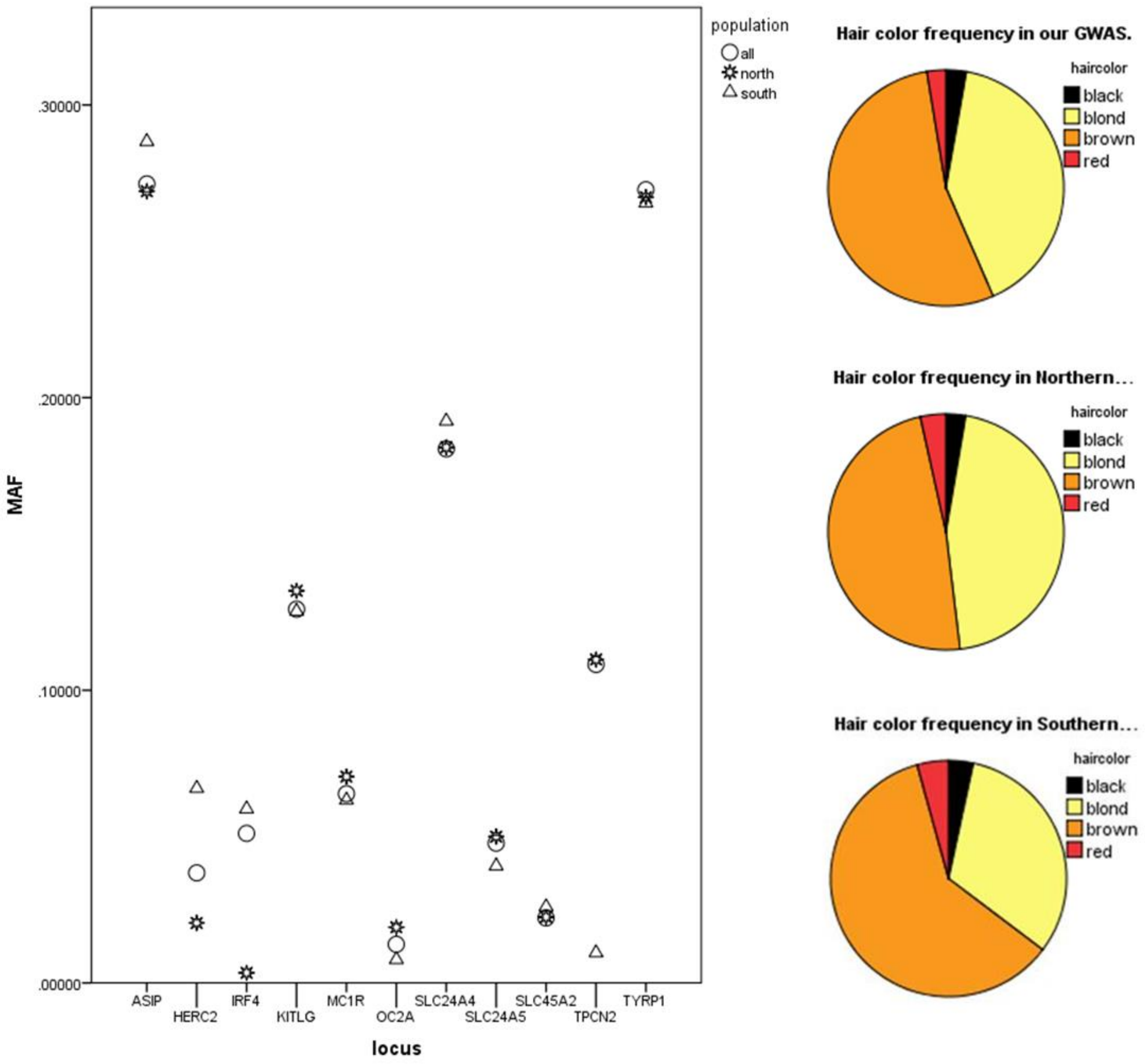

Figure S12. The minor allele frequency of the known hair color genes in relation to the hair color distribution, comparing the total GWAS sample to the 1000 PC1 based most northern individuals and 1000 PC1 based most southern individuals.

References

1. Falconer, D.S.; Mackay, T.F.C. *Introduction to Quantitative Genetics*, 4th ed.; Longman: Essex, UK, 1996.
2. Hutton, S.M.; Spritz, R.A. Comprehensive analysis of oculocutaneous albinism among non-hispanic caucasians shows that oca1 is the most prevalent oca type. *J. Investig. Dermatol.* **2008**, *128*, 2442–2450.
3. Seyedahmadi, B.J.; Rivolta, C.; Keene, J.A.; Berson, E.L.; Dryja, T.P. Comprehensive screening of the ush2a gene in usher syndrome type ii and non-syndromic recessive retinitis pigmentosa. *Exp. Eye Res.* **2004**, *79*, 167–173.
4. Abdellaoui, A.; Hottenga, J.J.; de Knijff, P.; Nivard, M.G.; Xiao, X.; Scheet, P.; Brooks, A.; Ehli, E.A.; Hu, Y.; Davies, G.E.; *et al*. Population structure, migration, and diversifying selection in the netherlands. *Eur. J. Hum. Genet.* **2013**, *21*, 1277–1285.
